# Effects of natural nitrite source from Swiss chard on quality characteristics of cured pork loin

**DOI:** 10.5713/ajas.19.0117

**Published:** 2019-05-27

**Authors:** Tae-Kyung Kim, Ko-Eun Hwang, Dong-Heon Song, Youn-Kyung Ham, Young-Boong Kim, Hyun-Dong Paik, Yun-Sang Choi

**Affiliations:** 1 Research Group of Food Processing, Korean Food Research Institute, Wanju 55365, Korea; 2 Department of Food Science and Biotechnology of Animal Resources, Konkuk University, Seoul 05029, Korea

**Keywords:** Swiss Chard, Natural Nitrite, Synthetic Nitrite, Meat, Cured Pork Loin

## Abstract

**Objective:**

This study was conducted to evaluate quality characteristics of cured pork loin with natural nitrite source from Swiss chard.

**Methods:**

Pork loin was cured in brine and the ratio of water and fermented Swiss chard (FSC) solution in the brine was changed by 4:0 (control), 3:1 (T1), 1:1 (T2), 1:3 (T3), 0:4 (T4), and pickled samples with 0.012% sodium nitrite (PC, positive control) and nitrite free brine (NC, negative control) were considered as the control.

**Results:**

The pH values of cured pork loins with FSC were decreased with increasing addition level of FSC. Cooking loss was not significantly different among all treatments. T4 had the lowest value in moisture content and lightness value and the highest value in curing efficiency. The redness value of T4 was not significantly different from that of PC in raw. After cooking, however, it was higher than that of PC. The yellowness value of cured pork loin added with FSC was increased with increasing level of FSC. Volatile basic nitrogen content of cured pork loin added with FSC was higher than PC and NC. Thiobarbituric acid reactive substance value of cured pork loin added with FSC was decreased with increasing FSC level. Residual nitrite level and shear force were increased with increasing FSC level. In the sensory evaluation, sensory score for flavor, off-flavor, chewiness, juiciness, and overall acceptability were not significantly different among all treatments. However, sensory score for color was increased when the concentration of FSC added to pork loin was increased.

**Conclusion:**

The FSC solution had a positive effect on redness and lipid oxidation. As shown by the results in protein deterioration and sensory, Swiss chard can replace sodium nitrite as natural curing agent.

## INTRODUCTION

In the past, people have used nitrite and nitrate salts to enhance the shelf stability, flavor, antimicrobial activities, and color of meat and meat products and direct addition of sodium nitrite have been used in curing meat and meat products [1]. Nitrite can develop color and restrain toxin production of pathogenic bacteria such as *Clostridium botulinum* of meat and meat products. Nitrite is converted into nitric oxide which form NO-Mb and nitrosohemochrome with myoglobin which is a pinkish color [2]. Despite these advantages, It is controversial issue which is addition of nitrate and nitrite to meat products due to carcinogenic potency of nitrite by forming N-nitrosamine with amines in meat products [3]. Because of this negative effect of nitrite, the development of natural and healthy meat product have been needed. The focus on meat products has moved to “natural” meat product and the concern about chemical curing agent such as nitrite and nitrate make that consumer felt constrained to pay for natural foods with growing interest about health care. Some researchers have tried to search for natural alternatives of nitrite from soil, water, and plants to replace synthetic nitrite [4].

Celery powder has been used as flavor enhancer and nitrate-reducing source to produce some fermented sausages since the 1950s. When celery powder is used in the curing process, it must be incubated to convert nitrate to nitrite prior to the heating process for suitable color formation [5]. However, Ballmer-Weber et al [6] reported allergic reaction to celery using double-blind placebo-controlled food challenges with raw-celery, cooked celery and celery spice, even after heating for 76 min at 100°C. Therefore, the use of celery on food needs to be watched for celery-allergic patients and celery must be substituted with non-allergenic materials.

Swiss chard (*Beta vulgaris cicla*) is a vegetable in the family of Chenopodiaceae. It can be cultivated easily in poor surroundings. Since 1000 B.C., Swiss chard has been used for nourishment by Mediterranean people. Swiss chard has antihypertensive and hypoglycaemic action and also possesses antioxidant components such as polyphenols, flavonoids, and vitexin [7]. According to Ninfali and Angelino [8], total phenols, flavonoids, and oxygen radical absorbance capacity of leaves of Swiss chard were 11.12±0.56 mg/g dry weight (DW), 7.92±0.39 mg/g DW, and 192.8±9.6 trolox equivalents μmol/g DW and nitrate in plant is formed by absorbed nitrogen. The mean nitrate content in Swiss chard is 568.5 to 3,407.4 ppm [9]. Therefore, Swiss chard can be a good source of nitrate which can be converted to nitrite for natural curing. However, no study has reported the effect of pre-converted Swiss chard on cured meat.

Therefore, the objective of this study was to assess the possibility of using pre-converted Swiss chard as a source of natural nitrite for cured meat without direct addition of sodium nitrite.

## MATERIALS AND METHODS

### Preparing natural nitrite source from Swiss chard and the processing of cured pork loin

Freeze-dried Swiss chard powder that was obtained from local market was diluted with distilled water, and 10% (w/v) Swiss chard solution was incubated with starter culture (S-B-61, Bactoferm, Chr. Hansen Inc., Gainesville, FL, USA) at 37°C for 24 h and starter culture contained only *Staphylococcus carnosus*. Fermented Swiss chard solution (FSC, pH 5.24, Commission Internationale de l’Eclairage [CIE] L*-value, 14.05; CIE a*-value, 2.03; CIE b*-value, 6.98; nitrite content, 322 ppm) was used immediately after incubation. Nitrite content in fermented Swiss chard was measured by diazo coupling method [10]. Formulation of cured pork loin was shown in Table 1. Fresh pork loin (*M. longissimus lumborum*) was obtained from a local processor postmortem 48 h. After trimming excessive fat and connective tissue, each loin was cut into 2.54 cm in thickness. After pickled pork loins in 40% brine in proportion to pork loin weight, samples were tumbled with a tumbler (MKR-150C, Ruhle GmbH., Grafenhausen, Germany) for 60 min at 4°C. Tumbled treatments were cured for 4 days in refrigerated room at 4°C [11]. After curing, cured pork loins were heated for 30 min at 75°C and cooled at room temperature (25°C) for 1 h.

### pH

Homogenate of cured pork loins (5 g in 20 mL distilled water) on raw and cooked were used for pH measurement. All determinations were performed in triplicates. The pH meter (Model 340, Mettler-Toledo GmbH, Greifensee, Switzerland) was used for measuring.

### Cooking loss

After heating for 30 min at 75°C, a sample was cooled to room temperature (25°C) for 1 h. Weight difference between before and after heating was calculated into a percentage.

### Moisture content

According to AOAC [12], moisture content (950.46B) of cooked samples was determined based on weight loss after 12 h of drying at 105°C in a drying oven (SW-90D, Sang Woo Scienectific Co., Bucheon, Korea).

### Color

Raw and cooked cured pork loins were sliced lengthwise. The internal surface color of samples was determined immediately. CIE L*-value, CIE a*-value, and CIE b*-value values were expressed as lightness, redness, and yellowness and a colorimeter (Minolta Chroma meter CR-210, Minolta Ltd., Tokyo, Japan; illuminate C, calibrated with a white plate, CIE L* -value, 97.83; CIE a*-value, 0.43; CIE b*-value, 1.98) was used.

### Curing pigments and total pigments

Hornesey’s method [13] was used to determine NO-heme (curing pigment) and total heme pigments (total pigment). Ten grams of ground sample was mixed with 80% (v/v) acetone for 5 min in reduced light to obtain curing pigment concentration. After filtering the solution through Whatman No. 1 filter paper (Whatman International, Maidstone, UK), absorbance of filtrate was measured at 540 nm using spectrophotometer (Optizen 2120 UV plus, Mecasys Co. Ltd., Daejeon, Korea) and NO-heme concentration was calculated by multiplying absorbance by 290. Total pigment concentration was obtained with 10 g minced sample and acidified 80% acetone. After mixing for 1 h, solution was filtered through Whatman No. 1 filter paper. Absorbance of filtrate was obtained at 640 nm using spectrophotometer and multiplied by 680 to determine total pigment concentration. Curing efficiency is the percentage of total pigment converted to curing pigment and it could be used as index of the degree of cured color fading.

### Thiobarbituric acid reactive substances value

Lipid oxidation was assessed in triplicates using the 2-thiobarbituric acid (TBA) method of Tarladgis et al [14] with minor modifications. After homogenize cooked sample with 97.5 mL of distilled water, a mixture was added with 2.5 mL of 4 N HCl and a few drops of an antifoam agent, silicone o/w (KMK-73, Shin-Etsu Silicone Co., Ltd., Seoul, Korea) into a distillation flask. After collect 50 mL distillate, a well mixture of 5 mL of the distillate and 5 mL of 0.02 M TBA in 90% acetic acid (TBA reagent) in capped tube heated in a boiling water bath at 100°C for 30 min and a blank was prepared with 5 mL of distilled water and 5 mL of TBA reagent. After cooling for 10 min at room temperature, the absorbance of reactant was measured at 538 nm with an UV/VIS spectrophotometer (Optizen 2120 UV plus, Mecasys Co. Ltd., Korea). Thiobarbituric acid reactive substances (TBARS) content was calculated from a standard curve (8 to 50 nmol) of malondialdehyde (MDA), freshly prepared by acidification of 1,1,3,3-tetraethoxy poppane. Reagents were obtained from Sigma (St. Louis, MO, USA). TBARS levels were expressed as MDA mg/meat kg.

### Volatile basic nitrogen content

Volatile basic nitrogen (VBN) content was assessed using the micro-diffusion method [15]. A 5 g of sample was homogenized with 45 mL of distilled water for 2 min at 8,000 rpm and then filtered through filter pater Whatman No. 1 (Whatman International, UK). One milliliter of the filtered sample solution and 1 mL of K_2_CO_3_ solution were placed on the outer section of a Conway micro-diffusion cell. One milliliter of 0.01 N H_3_BO_3_ and 50 μL of indicator (0.066% methyl red in ethanol: 0.066% bromocresol green in ethanol = 1:1) were placed on the inner section. Cells were incubated for 90 min at 37°C and then titrated with 0.02 N H_2_SO_4_ solution until a faint reddish color was produced.

### Residual nitrite level

According to the Diazo coupling method [10], residual nitrite level was determined. A 10 g of cured pork loin was blended with 150 mL pre-heated distilled water for 2 min. Before heating in a water bath at 80°C, 10 mL 0.5 N sodium hydroxide and 10 mL 12% ammonium thiosulfate were added into the blended solution. After cooling, 20 mL ammonium acetate buffer was added in the solution. The solution was then filled up to 200 mL with distilled water. After 10 min at room temperature, the sample was filtered through filter pater Whatman No. 1 (Whatman International, UK). A 1 mL of sulphanilamide solution, 1 mL N-(1-naphyhyl) ethylenediamine dihydrochloride reagent and 3 mL distilled water were added into 20 mL filtered solution. After 20 min at room temperature, the absorbance value at wavelength of 540 nm was read in a UV/VIS spectrophotometer (Optizen 2120 UV plus, Mecasys Co. Ltd., Korea). The residual nitrite content was calculated from the standard curve of nitrite solution.

### Warner-Bratzler shear force

Cooked samples used for the measurement of cooking loss were measured with a texture analyzer (TA-XT2*i*, Stable Micro Systems Ltd., Godalming, UK) using Warner-Bratzler shear attachment (V-type blade set). Prior to analysis, samples were prepared by sampling device into 1.5×1.5×5 cm (height× width×length) in the muscle fiber direction. Test speeds were set at 2 mm/s. Data were collected and analyzed from the maximum force required to shear force through each sample [16].

### Sensory evaluation

A trained ten-member panel consisting of researchers from the Department of Food Sciences and Biotechnology of Animal Resources at Konkuk University in Korea evaluated cured pork loins. Each treatment was evaluated in terms of interior color, flavor, off-flavor, chewiness, juiciness, and overall acceptability. Cured pork loin sample was cooked at 75°C for 30 min, cooled at room temperature for 30 min, and served randomly to the panelists. Sensory evaluations were performed under fluorescent lighting. Panelists were instructed to cleanse their palates between samples using water. Each sensory traits (interior color, flavor, off-flavor, chewiness, juiciness, and overall acceptability) were evaluated using a 9-point descriptive scale (1 = very undesirable, 9 = very desirable). Analysis was conducted using the descrptive test described by Bergara-Almeida and da Silva [17].

### Statistical analysis

The statistical analysis of all data was performed by IBM SPSS Ver. 20.0 (IBM, North Castle, NY, USA). The one-way analysis of variance and Duncan’s multiple range tests were used to find the differences among treatments (p<0.05). All means values were obtained from independent triple tests for each trait.

## RESULTS AND DISCUSSION

### pH, cooking loss, and moisture content

The pH, cooking loss, and moisture content of cured pork loin with natural nitrite source from Swiss chard and sodium nitrite are summarized in Table 2. The pH values of raw and cooked cured pork loins were decreased with increasing addition level of fermented Swiss chard solution (p<0.05). Kim et al [18] reported similar result that pH of cured meat with fermented spinach extract had lower pH values than cured meat with nitrite. Low pH value of fermented Swiss chard (pH 5.24) might result in the low pH of cured meat raw and cooked. The acidic pH of brine and cured meat plays an important role in enhancing the cured color formation, flavor and antimicrobial activity of cured meat because nitrite can be easily reduced to nitric oxide on acidic pH state [19]. Therefore, the decreasing pH value by fermented Swiss chard might have a positive effect on the quality characteristics of cured meat.

It was hypothesized that low pH of final product might have negative effect on cooking loss and moisture content. Although cooking loss of cured pork loins was not significantly different from each other (p>0.05), moisture content of cured meat was decreased significantly with increasing level of FSC in brine (p<0.05). It has been reported that myofibrils with higher pH value has higher moisture content than those with lower pH values because of the net charge on myofibrils affected by pH of meat [20,21]. Cooking loss can be affected by various causes such as salt, additives, size, shape and composition of cured meat [22]. However, no significant difference was observed between treatments (p>0.05). Krause et al [23] reported similar result that there is no significant difference in cooking yield of cured ham added with nitrite or pre-converted vegetable juice.

### Color and curing efficiency

Table 3 provides the lightness (CIE L*-value), redness (CIE a*-value), and yellowness (CIE b*-value) of the internal surface of raw and cooked cured meat. In general, cured meat has bright red color due to NO-myoglobin and nitrosohemochrome formed by nitric oxide with myoglobin on raw and cooked [2,24]. The internal surface color of raw cured meat was significantly different (p<0.05). Kim et al [18] reported similar result that redness value of cured meat with fermented spinach extract was increased with increasing level of fermented spinach extract. The redness values of control with 0.012% sodium nitrite and T4 were not significantly different (p>0.05) and redness value of T1 was lower than that of T4 (p<0.05). However, the redness of some treatments (T2 and T3) was not significantly different with negative control (NC) (p>0.05) and T1 has significantly lower redness value (p<0.05) compared with NC. It might be a deficient nitrite content in brine of treatment and typical color of FSC. While FSC permeated into pork loin in the curing process, yellowness values of raw treatments were increased and the lightness values decreased due to the color of FSC (CIE L*-value, 14.05; CIE a*-value, 2.03; CIE b*-value, 6.98). After cooking, the redness values of cured meat with FSC were increased with increasing level of FSC. The redness value of T4 was higher than that of positive control (PC) (p<0.05) and T3 was not significantly different compared with PC (p>0.05) because of the pinkish color of heat stable nitrosohemochrome formed after cooking. Sindelar et al [25] reported similar result that there was no significant difference in redness value between vegetable powder cured treatment and nitrite added control. This result demonstrated that FSC can be used as substitution of nitrite to form pinkish color of cooked cured meat. However, T1 and T2 had lower redness than NC (p< 0.05) because of the deficient nitrite content for the development of cured color pigment and the distinct color of FSC. Krause et al [23] reported that nitrite cured control had higher redness value than pre-converted vegetable juice powder cured treatment. In the natural curing, the incubation condition such as vegetable concentration and temperature are important for the conversion of nitrate to nitrite to develop the redness of cured meat [26]. Therefore, T3 and T4 were suitable for substitution of curing color enhancer.

Curing pigment, total pigment, and curing efficiency was shown in Table 3. There were significantly different by addition level of FSC and nitrite concentration. T4 had the highest value in curing pigment and curing efficiency (p<0.05) and curing pigment concentration was decreased with decrease level of FSC. The NC had the lowest value in curing pigment and curing efficiency (p<0.05). However, significant difference was not observed between NC and T1 in curing pigment and curing efficiency (p>0.05). Low nitrite concentration of T1 might lead this result that deficient curing pigment formed and T2 also had no significant difference between NC in curing pigment (p>0.05). T3 had a higher curing pigment than NC (p<0.05) and there were no significant different between curing pigment of PC and T3 (p>0.05). According to Terns et al [26], total pigment concentration depended on differences of raw materials. Therefore, curing efficiency might be a useful index of cured color fading because concentration can be differed by characteristics of materials. In general, the curing efficiency of well cured meat excess 80% [27]. In this study, PC, T3, and T4 had a high curing efficiency that excessed 80% and when compared with redness value and curing efficiency, the tendency of redness and curing efficiency was similar.

### Thiobarbituric acid reactive substances and volatile basic nitrogen

**Figure 1:**
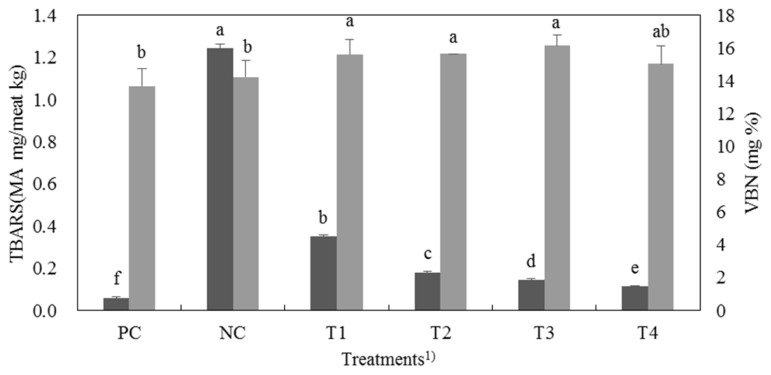
TBARS ( 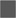) and VBN ( 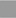) values of cured pork loin with natural nitrite source from FSC. TBARS, Thiobarbituric acid reactive substances; VBN, volatile basic nitrogen; FSC, fermented Swiss chard; MDA, malondialdehyde. ^1)^ PC, positive control, 120 ppm nitrite; NC, negative control, nitrite-free; T1, FSC solution:water = 10:30; T2, FSC solution:water = 20:20; T3, FSC solution:water = 30:10; T4, FSC solution:water = 40:0. ^a–f^ Different letters on top of the column mean significant difference between treatments p<0.05).

Figure 1 provides TBARS and VBN values of cooked cured pork loins. PC had the lowest TBARS and VBN values among all treatments (p<0.05). Choi et al [28] reported that lipid oxidation can affect the quality characteristics of meat products such as color, flavor, and nutritious value. With increasing levels of FSC, there was a decreasing tendency in TBARS value (p<0.05) because the amount of nitrite of FSC was increased. Sindelar et al [25] reported that natural nitrite from vegetable powder worked as an antioxidant. Tsoukalas et al [29] also reported that vegetable powder has antioxidant activity equivalent to nitrite. They reported that fermented sausage containing freeze-dried leek powder had similar TBARS value to that of the control containing 150 ppm nitrite. Furthermore, the antioxidant capacity of Swiss chard might inhibit lipid oxidation of cured meat. Ninfali and Angelion [8] reported that Swiss chard has abundant vitamins, phenols, and flavonoids that can prevent lipid oxidation.

Meat protein can be decomposed by some enzymes and microorganisms during the storage period. Protein deterioration could be indicated by VBN content [15]. The VBN content of cured meat with FSC was higher than that of PC or NC (p<0.05). Kim et al [18] reported similar result that the VBN values cured meat with fermented spinach solution was higher than cured meat with nitrite. This result might be caused by starter culture in FSC.

### Residual nitrite content

**Figure 2:**
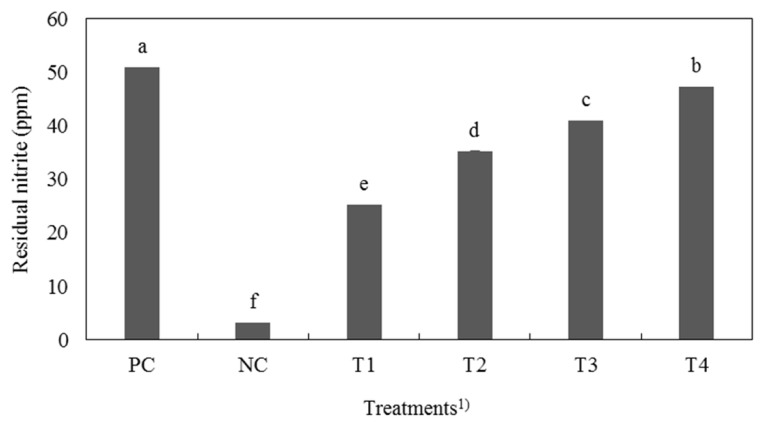
Residual nitrite contents of cured pork loin with natural nitrite source from fermented Swiss chard (FSC). ^1)^ PC, positive control, 120 ppm nitrite; NC, negative control, nitrite-free; T1, FSC solution:water = 10:30; T2, FSC solution:water = 20:20; T3, FSC solution:water = 30:10; T4, FSC solution:water = 40:0. ^a–f^ Different letters on top of the column mean significant difference between treatments (p<0.05).

Residual nitrite content of cooked cured pork loin is presented in Figure 2. Residual nitrite contents of cured pork loin were significantly different among all treatment (p<0.05). According to Kim et al [18], residual nitrite content of cured meat with fermented spinach was lower than that of cured meat with sodium nitrite. Residual nitrite content of cured meat with FSC also was lower than that of PC. Acidic pH and abundant antioxidant during curing process can increase the rate of curing color formation and decrease residual nitrite content because the acidic pH state and antioxidant such as phenols and flavonoids in meat and meat products can easily deplete nitrite to nitric oxide [29–32]. Therefore, the low pH and antioxidant components of FSC might deplete nitrite in cured meat containing FSC, whereas PC had highest residual nitrite content because it has higher pH value than the others (p<0.05). In general, residual nitrite content of cooked meat is decreased about 65% during the heating [2]. Because the combination of heat, acidic pH, and antioxidant depleted nitrite in FSC treatment, treatments with FSC had lower residual nitrite levels less than PC.

### Warner-Bratzler shear force

**Figure 3:**
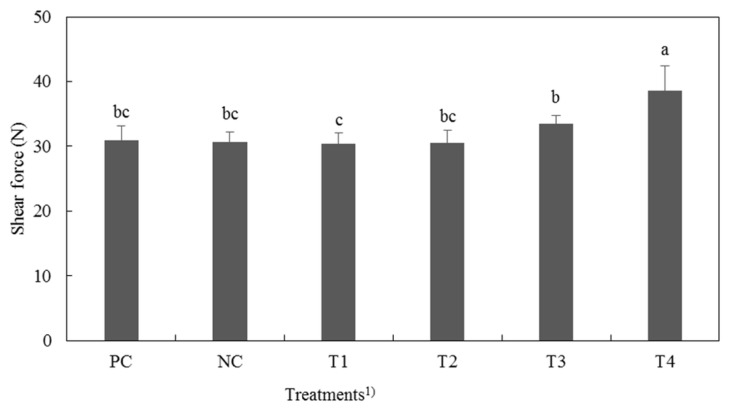
Warner-Bratzler Shear force contents of cured pork loin with natural nitrite source from fermented Swiss chard (FSC). ^1)^ PC, positive control, 120 ppm nitrite; NC, negative control, nitrite-free; T1, FSC solution:water = 10:30; T2, FSC solution:water = 20:20; T3, FSC solution:water = 30:10; T4, FSC solution:water = 40:0. ^a–c^ Different letters on top of the column mean significant difference between treatments (p<0.05).

Figure 3 provides Warner-Bratzler shear force (WBSF) values of cooked cured pork loins. WBSF values of cooked pork loin were significantly different (p<0.05). T4 showed the highest WBSF value among all treatments while T1 had the lowest WBSF value (p<0.05). The Shear force and pH value of meat are generally in inverse proportion [17]. Ruiz-Ramírez et al [33] showed that low pH cured meat had higher hardness value than the high pH cured meat. In general, pH of meat affected net charge of meat and structure. Meat with higher pH has higher moisture content than lower pH meat. According to Barlocco et al [34], moisture content and WBSF of pork muscle had negative correlations. Therefore, the lower the pH treatment had, the higher the value of WBSF (p<0.05).

### Sensory evaluation

Table 4 presented the evaluation score of the cured pork loin in sensory test. Sensory scores for flavor, off-flavor, chewiness, juiciness, and overall acceptability were not significantly different among all treatments (p>0.05). In instrumental measurement, shear force and moisture contents had different values among each treatment. However, chewiness and juiciness were no significantly different in sensory evaluation. There was no significant difference in color intensity among NC, T1, T2, and T3 (p>0.05), whereas the score for reddish color of PC and T4 were higher than other treatments (p<0.05). Sindelar et al [25] reported that the characteristic aroma of vegetable juice would have negative impact on meat. Tsoukalas et al [29] also reported that lower vegetable level in meat product had higher sensory scores for flavor. In this study, no significant difference was found in the overall acceptability and flavor among all treatments (p>0.05). This means that the color and flavor of FSC had no significant impact on the overall acceptability of cured pork loin containing FSC.

## CONCLUSION

In conclusions, quality characteristics of cured pork loin with natural nitrite source from Swiss chard were investigated in this. When the addition level of FSC was increased, the redness value of cured meat was increased and lipid oxidation was decreased. Residual nitrite contents of cured pork loins containing FSC were much lower than nitrite added treatment. Furthermore, the addition of FSC did not have any impact on overall acceptability. Hence, pre-converted Swiss chard could be used as natural curing agent for cured pork loin and substitute synthetic nitrite.

## Figures and Tables

**Table 1:** Formulation of cured pork loin with natural nitrite source from fermented Swiss chard (FSC)

Ingredients	Treatments1)					

	PC	NC	T1	T2	T3	T4
Pork loin (%)	100	100	100	100	100	100
Water (%)	40	40	30	20	10	0
Fermented Swiss chard solution (%)	0	0	10	20	30	40
Salt (%)	1.5	1.5	1.5	1.5	1.5	1.5
Nitrite (ppm)	120	0	32.2	64.4	96.6	128.8

1) PC, positive control, 120 ppm nitrite; NC, negative control, nitrite-free; T1, FSC solution:water = 10:30; T2, FSC solution:water = 20:20; T3, FSC solution:water = 30:10; T4, FSC solution:water = 40:0.

**Table 2:** The pH value, cooking loss and moisture content of cured pork loin with natural nitrite source from fermented Swiss chard (FSC)

Traits	Treatments1)					

	PC	NC	T1	T2	T3	T4
pH value						
Raw	5.81±0.01^a^	5.81±0.02^a^	5.81±0.01^a^	5.80±0.01^bc^	5.79±0.01^bc^	5.74±0.01^c^
Cooked	6.08±0.02^a^	5.97±0.03^b^	6.08±0.01^a^	5.98±0.01^b^	5.97±0.02^b^	5.94±0.02^c^
Cooking loss (%)	24.55±1.74	24.65±2.04	25.92±3.27	25.96±3.16	25.74±1.75	25.02±2.32
Moisture content (%)	68.24±0.63^a^	68.08±0.30^a^	67.01±0.47^ab^	67.11±0.46^ab^	66.22±0.16^bc^	65.43±1.37^c^

1) PC, positive control, 120 ppm nitrite; NC, negative control, nitrite-free; T1, FSC solution:water = 10:30; T2, FSC solution:water = 20:20; T3, FSC solution:water = 30:10; T4, FSC solution:water = 40:0.

a–c Means within a row with different letters are significantly different (p<0.05).

**Table 3:** Color characteristics and curing efficiency of cured pork loin with natural nitrite source from fermented Swiss chard (FSC)

Traits	Treatments1)					

	PC	NC	T1	T2	T3	T4
Raw						
L*	51.77±1.52^b^	63.96±0.83^a^	44.73±1.52^d^	36.22±1.05^e^	38.27±0.69^d^	33.60±0.48^f^
a*	8.60±0.84^a^	6.11±0.64^b^	5.56±0.67^c^	6.80±0.62^b^	6.49±0.27^b^	8.37±0.22^a^
b*	4.88±0.79^c^	1.82±0.50^e^	4.65±1.48^c^	3.32±0.32^d^	6.97±0.96^b^	9.56±0.90^a^
Cooked						
L*	72.50±0.48^b^	69.76±0.24^c^	74.91±0.33^a^	68.71±0.54^d^	68.58±0.48^d^	66.71±0.35^e^
a*	8.47±0.33^b^	5.80±0.14^c^	5.26±0.27^d^	5.32±0.30^d^	8.62±0.05^b^	9.08±0.48^a^
b*	5.55±0.07^e^	7.89±0.20^c^	8.42±0.25^b^	8.88±0.15^a^	8.04±0.10^c^	7.30±0.14^d^
Curing pigment (ppm)	33.93±3.94^b^	19.65±1.66^c^	20.16±0.77^c^	18.85±1.97^c^	31.97±1.16^b^	40.46±3.99^a^
Total pigment (ppm)	39.44±0.79^b^	37.06±1.18^c^	32.47±0.65^e^	28.90±0.39^f^	35.70±1.17^d^	44.88±0.96^a^
Curing efficiency (%)	86.03±9.81^a^	53.07±4.98^c^	62.09±2.57^bc^	65.24±6.99^b^	89.56±1.19^a^	90.19±9.41^a^

1) PC, positive control, 120 ppm nitrite; NC, negative control, nitrite-free; T1, FSC solution:water = 10:30; T2, FSC solution:water = 20:20; T3, FSC solution:water = 30:10; T4, FSC solution:water = 40:0.

a–f Means within a row with different letters are significantly different (p<0.05).

**Table 4:** Sensory properties of cured pork loin with natural nitrite source from fermented Swiss chard (FSC)

Traits	Treatments1)					

	PC	NC	T1	T2	T3	T4
Color	8.25±0.96^a^	6.00±0.82^c^	6.25±0.50^c^	6.75±1.26^bc^	6.75±0.5^bc^	7.75±0.5^ab^
Flavor	7.50±1.05	7.00±0.89	6.50±1.22	6.50±0.55	6.67±1.03	6.33±1.03
Off flavor	7.50±1.05	6.33±1.37	6.83±1.33	6.67±0.82	7.00±0.89	6.50±1.05
Chewiness	7.33±0.52	7.17±0.75	7.00±1.26	6.83±0.75	7.17±0.98	7.17±0.75
Juiciness	7.17±0.41	6.67±0.82	6.67±1.37	6.67±0.52	6.67±0.52	6.50±0.84
Overall acceptability	7.50±0.84	6.17±0.41	6.67±1.21	6.40±0.55	6.83±0.98	6.83±0.75

9-point descriptive scale (1 = very undesirable, 9 = very desirable) was used for sensory evaluation

1) PC, positive control, 120 ppm nitrite; NC, negative control, nitrite-free; T1, FSC solution:water = 10:30; T2, FSC solution:water = 20:20; T3, FSC solution:water = 30:10; T4, FSC solution:water = 40:0.

a–c Means within a row with different letters are significantly different (p<0.05).
